# Deep Learning and Noninvasive Sensors for Detecting Physiological Dysregulation: A Scoping Review

**DOI:** 10.1007/s10916-025-02332-7

**Published:** 2026-01-30

**Authors:** Mariana González Garcés, Jerónimo Cárdenas Montoya, María Isabel Peña Martínez, Juanita Valencia García, Erwin Hernando Hernández Rincón

**Affiliations:** 1 Primary Care Physician, Faculty of Medicine, Universidad de La Sabana, Chía, Colombia; 2 Department of Family Medicine and Public Health, Universidad de La Sabana, University Campus Puente del Común, Km 7, Autopista Norte, Chía, Colombia

**Keywords:** Acute pain, Artificial intelligence, Deep learning, Early detection, Hemodynamic instability, Intensive care units, Non-invasive sensors, Physiological monitoring, Physiological stress

## Abstract

Early detection of pain, stress, or hemodynamic instability is key to preventing serious clinical events. In recent years, non-invasive sensors and deep learning algorithms have gained relevance as tools for accurate and continuous monitoring. To map and synthesize the scientific evidence on the use of non-invasive multimodal sensors combined with deep learning algorithms for the early detection of physiological dysregulation states, including pain, stress, and hemodynamic deterioration, in patients over 13 years of age in clinical settings. A scoping review was conducted following the JBI and PRISMA-ScR guidelines. We included studies published between 2019 and 2025 in English or Spanish, identified through three databases and a secondary search. Twenty-seven studies were analyzed after duplicate removal and screening. Deep learning algorithms applied to electroencephalograms, electrocardiograms, photoplethysmography, and facial image signals showed high accuracy in predicting clinical events such as pain or hypotension. China and Australia had the highest number of included studies (*n* = 3), followed by South Korea, the United States, and Greece (*n* = 2 each). Retrospective and experimental designs predominated, with applications in intensive care units, operating rooms, and emergency rooms. These technologies represent an emerging strategy with high potential to improve early detection in clinical practice. However, further validation in real-world environments, optimization of implementation methods, and evaluation of their clinical impact are still needed.

## Introduction

Pain and nociception are closely related but not identical concepts. Pain is a subjective experience that encompasses both sensory and emotional dimensions and is associated with actual or potential tissue damage, whereas nociception refers to the neurophysiological processes of detecting noxious stimuli without the need for consciousness [3, 5]. This distinction is especially relevant in clinical contexts such as general anesthesia, where awareness is absent and nociception, rather than pain, better describes the organism’s responses [2, 8]. Understanding this difference is critical to improve monitoring and management in situations where subjective assessment is not possible, including sedated, anesthetized, or critically ill patients [9, 11, 13].

Over the past two decades, noninvasive technologies for clinical monitoring have transformed the early detection of physiological alterations. Devices such as electrocardiograms (ECG), photoplethysmography (PPG), electroencephalography (EEG), and electrodermal activity (EDA) sensors enable continuous and automated measurement of physiological variables related to pain, stress, and hemodynamic instability [6, 7, 10, 12]. However, traditional analytical methods often fail to identify complex nonlinear patterns within these signals [13]. In this context, deep learning, a subfield of artificial intelligence, has emerged as a powerful tool for biomedical data analysis due to its capacity to extract hierarchical representations and discover subtle relationships across multimodal data [1, 14].

Deep learning–based monitoring systems have demonstrated the ability to reduce false positives, anticipate cardiovascular collapse, and minimize alarm fatigue while supporting timely interventions [15, 16]. Furthermore, the integration of multimodal physiological data such as EEG, ECG, and PPG has shown potential not only to enhance anesthetic management but also to improve patient safety in intensive care and surgical environments [17–19].

Currently, stress detection is based on physiological signals such as ECG, electrodermal activity, electromyography, respiratory rate, and body temperature. However, models relying on a single source of information, particularly indicators of autonomic nervous system activity, show limitations because of interference from non-nociceptive factors. Moreover, isolated EEG signals may not accurately reflect nociceptive state. Multimodal approaches that integrate various sources, such as EEGs, ECGs, and PPGs, offer a more comprehensive and robust assessment of pain-related physiological processes [32, 34, 35].

Given this background, it is appropriate to conduct a scoping review to map and describe the current evidence on the use of multimodal sensors combined with deep learning algorithms for the early detection of signs related to pain, stress, or hemodynamic impairment. This review aims to identify gaps in the literature, emerging trends, and opportunities for future clinical applications.

## Methodology

A scoping review was conducted to explore and characterize the available evidence on the use of noninvasive physiological sensors combined with deep learning algorithms in clinical settings. This method is appropriate for mapping emerging scientific fields, identifying key concepts, and summarizing existing evidence. The review followed the Joanna Briggs Institute guidelines for scoping reviews, and the protocol was registered on the Open Science Framework platform (osf.io).

The review question was structured using the PCC (Population, Concept, Context) framework: *What is the current scientific evidence regarding the use of noninvasive multimodal sensors combined with deep learning algorithms to detect early physiological dysregulation states*,* including pain*,* stress*,* and hemodynamic deterioration*,* in patients older than 13 years in clinical settings?*

### Search Strategy and Data Collection

A comprehensive search was conducted in PubMed, Scopus, and Web of Science. These databases were selected because of their broad coverage in biomedical and computational sciences. The search included studies published between 2019 and 2025 in English or Spanish.

The search strategy incorporated MeSH terms and free-text keywords such as *deep learning*, *noninvasive sensors*, *pain detection*, *stress monitoring*, and *hemodynamic instability*, combined with the Boolean operators AND and OR. A secondary manual search was performed by reviewing the reference lists of all articles selected in the initial phase. This snowball strategy yielded additional eligible studies. The complete database search strategies are summarized in Table 1.

**Tabla 1:** Search strategies

Database	Search algorithm
**PubMed**	“Pain “OR “Stress Physiological” OR “Hemodynamic Processes” OR “pain” OR “stress” OR “hemodynamic” OR “hemodynamic deterioration” AND “Deep Learning” OR “Machine Learning” OR “Artificial Intelligence” AND “Sensors” AND “Sensors”. OR “Monitoring Physiologic” AND “Patients”.
**Scopus**	“Pain “OR “Stress Physiological” OR “Hemodynamic Processes” OR “pain” OR “stress” OR “hemodynamic” OR “hemodynamic deterioration” AND “Deep Learning” OR “Machine Learning” OR “Artificial Intelligence” AND “Sensors” AND “Sensors”. OR “Monitoring Physiologic” AND “Patients”.
**Web of Science**	“Pain” AND “Deep Learning” AND “Patients” OR “hemodynamic deterioration” AND “Artificial intelligence”.

All retrieved records were managed using the Rayyan platform, which facilitated duplicate removal and allowed independent screening by two reviewers.

The final search was conducted on **January 15**,** 2025**.

## Selection of Studies

**Fig. 1:**
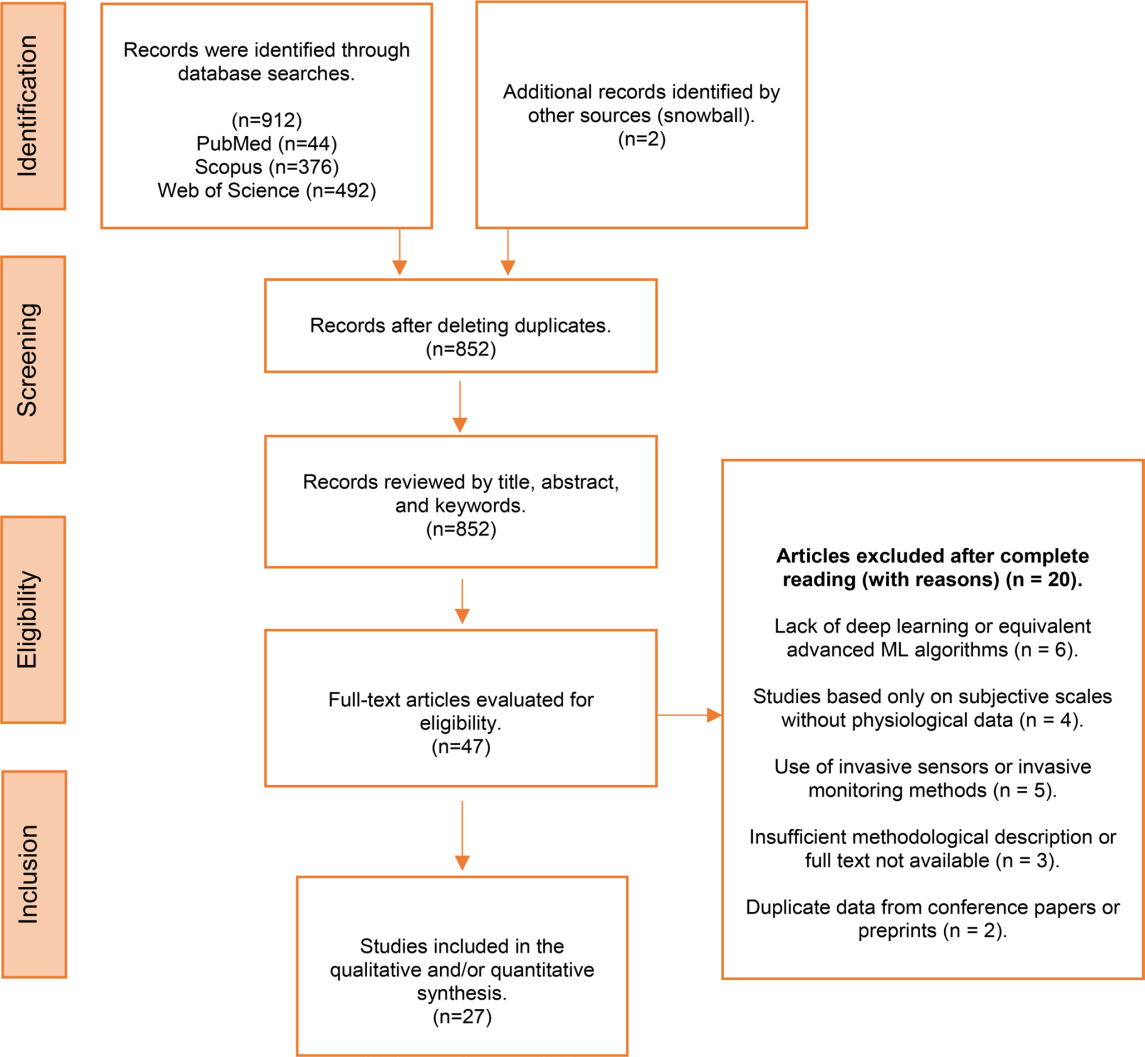
PrismaScR

The initial search identified **912** records from the databases, and **2** additional studies were found through snowballing. After removing **62** duplicates, **852** unique records were screened by title and abstract. From these, **47** articles were selected for full-text review. A total of **27** studies met the inclusion criteria and were included in the final synthesis. The study selection process is summarized in Fig. 1.

During full-text assessment, **20** studies were excluded for the following reasons:

- absence of deep learning or advanced machine learning algorithms (*n* = 6);
- reliance solely on subjective instruments or questionnaires without physiological data (*n* = 4);
- use of invasive sensors or invasive monitoring techniques (*n* = 5);
- insufficient methodological description or unavailable full text (*n* = 3);
- duplicate data reported in conference abstracts or preprints later published in included journals (*n* = 2).

## Inclusion and Exclusion Criteria

Included studies met the following criteria: original research (quantitative, qualitative, or mixed-methods), systematic reviews, or scoping reviews published between 2019 and 2025 in English or Spanish. Eligible studies used noninvasive physiological sensors—such as EEG, ECG, PPG, capnography, electrodermal activity, or other biomedical signals—combined with deep learning algorithms for the early detection of pain, physiological stress, or hemodynamic deterioration in patients older than 13 years. Clinical contexts included intensive care units, operating rooms, emergency departments, and general hospital wards.

Studies were also included if they addressed at least one of the following domains: pain, physiological stress, or hemodynamic deterioration. These conditions were analyzed jointly because they share overlapping physiological pathways, monitoring strategies, and deep learning processing pipelines.

Exclusion criteria included preclinical or animal studies, publications without available full text, articles in languages other than English or Spanish, and nonscientific literature such as editorials, letters to the editor, comments, or isolated case reports.

## Data Extraction

Data were extracted using a structured Excel matrix designed for this review. The matrix was aligned with the review objectives and validated by the research team prior to data extraction.

The extracted variables included: authors, year of publication, country, study design, clinical setting, population characteristics, noninvasive sensors used, deep learning algorithms applied, main outcomes, benefits, and reported limitations.

The reviewers independently extracted the data, and disagreements were resolved through discussion to ensure rigor and consistency.

## Data Analysis and Synthesis

Extracted information was organized into a synthesis table summarizing the key characteristics of the included studies. A narrative synthesis was performed, grouping the findings according to clinical context (e.g., intensive care unit, operating room, emergency department), type of sensor used (e.g., EEG, ECG, PPG, facial recognition, acoustic signals), and clinical target (pain, stress, or hemodynamic deterioration).

The synthesis also examined methodological approaches, clinical applications, technological developments, and reported limitations. Finally, the analysis identified existing evidence gaps, emerging research needs, and opportunities for future validation and clinical implementation.

## Results

A total of **27 studies** were included in this scoping review. These studies were published between 2019 and 2025 and evaluated the application of deep learning algorithms to noninvasive physiological signals for early detection of pain, physiological stress, or hemodynamic deterioration.

**Fig. 2:**
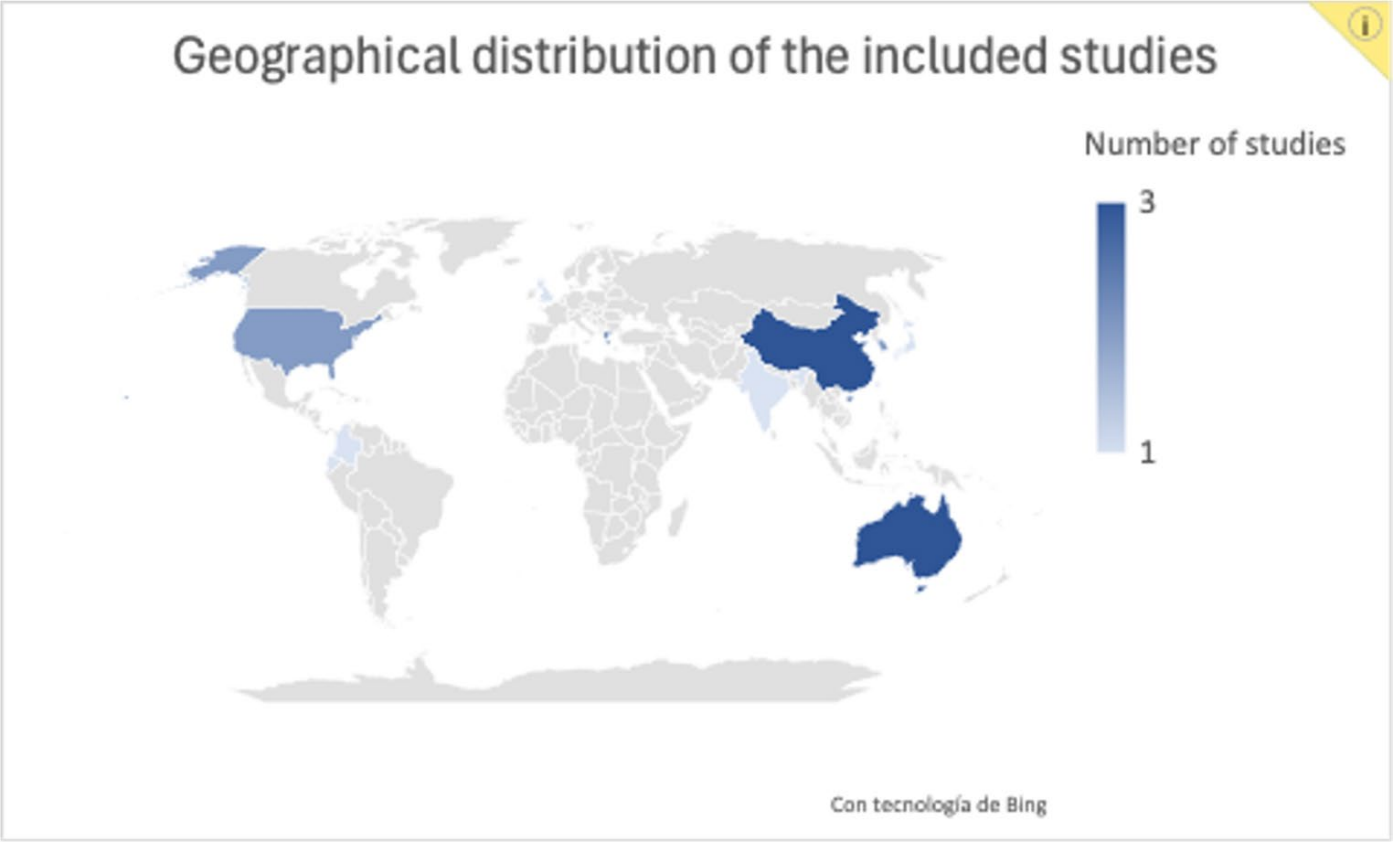
Geographical distribution of the included studies. China and Australia had the highest number of included studies (n = 3 each). South Korea had two included studies (n = 2), as did the United States and Greece. Other countries represented by a single study were India, Taiwan, Egypt, Japan, Colombia, Ecuador, and the United Kingdom. Additionally, several articles involved international collaborations, particularly between the United States, Saudi Arabia, and Italy

China and Australia contributed the highest number of studies (*n* = 3 each), followed by South Korea, the United States, and Greece (*n* = 2 each). Countries represented by a single study included India, Taiwan, Egypt, Japan, Colombia, Ecuador, and the United Kingdom. The geographical distribution of the included studies is shown in Fig. 2.

Regarding study design, **experimental studies** were the most frequent (*n* = 11), followed by **retrospective studies** (*n* = 9), **systematic reviews and meta-analyses** (*n* = 3), **observational studies** (*n* = 2), **one prospective study**, and **one Delphi study**. This distribution reflects a balance between exploratory approaches and clinical validation efforts. The most commonly used architectures included convolutional neural networks, long short-term memory networks, bidirectional LSTM networks, transformer-based models, and hybrid deep learning systems.

The physiological signals analyzed across studies included EEG, ECG, PPG, capnography, acoustic signals, facial images, and various multimodal combinations integrating two or more physiological or behavioral sources.

As summarized in Table 2, several studies reported high predictive performance. Jeong et al. [2] demonstrated AUROC values of 0.917 in development and 0.833 in external validation using noninvasive inputs such as NIBP, ECG, PPG, and BIS. Wu et al. [3] developed a pain classifier for critically ill patients with an accuracy of 85.9%. Bargshady et al. [7] achieved accuracies above 89% in multiclass pain classification using convolutional and recurrent architectures applied to facial images.

**Tabla 2:** Summary table

Author(s)/Year	Methods/Tools Used	Key Findings	Limitations
Hughes JA et al. [1]	Transformer applied to nursing notes	Identified pain in 55.16% of clinical presentations	Possible variability in documentation quality
Jeong H et al. [2]	NIBP, ECG, PPG, BIS, capnography	AUROC 0.917 (development) and 0.833 (external validation); good sensitivity and NPV	No prospective validation
Wu CL et al. [3]	CNN and BiLSTM	Multiclass accuracy 55.9%; binary 85.9%; video models up to 88%	Single-center study; small sample size
Jeddah D et al. [4]	Automated rule-based EHR analysis	Respiratory sensitivity 82.4%, specificity 92.1%; hemodynamic sensitivity 91.3%, specificity 84.9%	Retrospective labeling; data quality bias
Huo J et al. [5]	CNN, ResNet, VGG applied to facial images	Average AUC 0.86; accuracy 83.1%	Databases not representative of real clinical pain
Gkikas S et al. [6]	CNN, ResNet, VGG; multimodal and unimodal data	Mean AUC 0.86; accuracy 83.1%	Insufficient sociodemographic diversity
Bargshady G et al. [7]	VGGFace, PCA, CNN + RNN	Accuracy > 89%; AUROC 0.93	Not tested in real-world clinical settings
Solares GJ et al. [8]	Hypotension Prediction Index integrated into protocol	Fewer complications and reduced hospital stay by 2 days	Single-center; needs prospective validation
Jian Z et al. [9]	Autoencoder + Gaussian mixture model	Identified four hypotension endotypes; robust across validation cohorts	Possible population variability
Gutiérrez R et al. [10]	CNN for facial gestures + acoustic analysis	Multimodal approach outperformed unimodal methods	Limited to one country; homogeneous population
Buitrago S et al. [11]	CNN 1D + kernel spectral connectivity	Accuracy ~ 90%; AUC 0.91	Limited to young, homogeneous population
Semwal A et al. [12]	Compact CNN fusion for facial appearance and shape	Effective for pain classification and intensity estimation	Simulated dataset; lacking real-world validation
Wu F et al. [13]	CNN + BiLSTM + attention	Accuracy close to 90%	Simulated environment with young population
Bouazizi M et al. [14]	CNN, DNN, transformer	Pain/no pain accuracy 91.16%	No validation in real patients
Schmitzberger FF et al. [15]	ECG, HRV, HR morphology	Sensitivity 96.9%; specificity 79%; AUC 0.90	Retrospective; lacks prospective validation
Holder AL et al. [16]	Delphi panel; Hemodynamic Stability Index	Identified 19 outcomes associated with HSI	Variability between institutions; lack of standard metrics
Kim J et al. [17]	Recurrent neural network	Outperformed conventional warning systems (AUC 0.947)	No validation with real-time physiological signals
Gkikas S et al. [18]	Transformer for multimodal fusion	Accuracy 90.5% for pain classification	No validation with real-time physiological signals
Nazeer M et al. [19]	Oxygenation, HR, GSR; Raspberry Pi processing	Random Forest accuracy 87.26%; real-time stress detection	Classical ML; lacks deep learning
El-Tallawy SN et al. [20]	Facial recognition, EEG, HRV, wearable sensors	Good potential for objective pain assessment	No standardization in pain measures; ethical concerns
Jean WH et al. [21]	ANI, CNN, RNN, LSTM, BiLSTM, CNN-LSTM	CNN-LSTM showed best performance for pain scores	Limited sample; restricted to one type of surgery
Fernández Rojas et al. [22]	EDA, ECG, temperature sensors	High accuracy in pain level classification	Needs validation in larger real-world populations
Jaber D et al. [23]	SHAP explainability method	High prediction accuracy; increased interpretability	Experimental; not tested clinically
Yin Q et al. [24]	Signal analysis + LSTM	> 90% accuracy for anesthesia and consciousness	Small sample size; need for quantitative studies
Omar MT et al. [25]	EEG, PPG, ECG during surgery	Signal fusion improved pain assessment	Limited number of subjects
Park C et al. [26]	Random Forest + ECG, HR, BP, SpO2	AUC up to 0.89; detected delirium 4 h earlier	Retrospective labels; potential bias
Nerella S et al. [27]	Facial video in ICU; transformer networks; action units	Feasible for continuous pain monitoring	Dependent on video quality and manual annotations

Multimodal approaches showed important advantages over single-signal models. Gutiérrez et al. [10] and Gkikas et al. [6] combined facial and acoustic information and reported improved diagnostic sensitivity for pain and stress detection, with accuracies between 83 and 90% depending on architecture and signal combination. Jian et al. [9] identified physiologic hypotension endotypes using autoencoders and Gaussian mixture modeling with reproducibility across independent cohorts. Jeddah et al. [4] achieved 91.3% sensitivity and 84.9% specificity for detecting respiratory and hemodynamic deterioration through automated analysis of electronic clinical records. Kim et al. [17] and Park et al. [26] demonstrated early warning capabilities for cardiac arrest or delirium hours before clinical detection, supporting more timely intervention in intensive care units.

Most studies focused on adult patients in contexts where subjective evaluation is difficult or impossible, including sedated, anesthetized, or intubated individuals. Signals derived from neural and cardiovascular activity enabled the identification of patterns not detectable through traditional assessments. Adaptive deep learning methods improved accuracy compared with systems based on fixed physiological thresholds, allowing the detection of subtle changes in clinical status.

However, the included studies also highlighted important contextual limitations. In controlled clinical environments, confounding factors such as sedation level, autonomic tone alterations, and mechanical ventilation can modify physiological patterns, potentially reducing the generalizability of trained models. In semi-ambulatory or outpatient settings, signals are influenced by physical activity, environmental noise, and emotional state, requiring different preprocessing and modeling strategies. Recent studies using wearable sensors combined with deep learning demonstrated promising performance in real-world monitoring of pain and stress [10, 17, 20].

The narrative synthesis identified seven thematic categories that organize the included studies: type of sensors used, model architecture, physiological signal analyzed, clinical purpose, implementation environment, methodological quality, and ethical or regulatory considerations. These categories helped map methodological approaches, identify common applications, and highlight main evidence gaps. These thematic categories and their associated references are summarized in Table 3.

**Tabla 3:** Table of categories

Category	Description	References
**Detection Methods and Tools**	EEG, ECG, ABP, PPG, heart rate, facial images, voice, and combined multimodal data. Deep learning models used included CNN, BiLSTM, transformers, autoencoders, and hybrid architectures. Several studies achieved AUROC values above 0.90.	[15, 17, 22, 25, 28]
**Populations Studied**	Adults in postoperative care, ICU, and emergency settings; some studies included healthy volunteers. Many studies focused on sedated, anesthetized, or intubated patients where subjective assessment is not feasible.	[11, 14, 18, 20]
**Clinical Contexts of Application**	Intensive care units, operating rooms, emergency departments, and intraoperative monitoring. Applications include predicting hypotension, monitoring anesthesia depth, and detecting pain without requiring patient communication.	[13, 16, 23]
**Clinical Outcomes and Effectiveness**	Early detection of events such as hypotension (up to 5 min before onset); pain detection and stress recognition with accuracies between 80–95%. Multimodal approaches demonstrated improved diagnostic performance compared with unimodal models.	[9, 12, 19, 21]
**Barriers and Limitations**	Limited availability of large labeled datasets, bias in training samples, lack of external validation, heterogeneous study designs, expensive technologies, and reduced explainability in several models.	[10, 17, 20, 24]
**Recommendations**	Integrate AI into continuous monitoring, develop explainable models, conduct multicenter studies, validate algorithms in real-time environments, and train clinical personnel to promote safe implementation.	[8, 14, 22]
**Gaps and Future Research**	Need for studies in pediatric and geriatric populations, lack of longitudinal validation, need for stronger ethical and regulatory frameworks, and limited availability of diverse datasets for cross-validation.	[6, 18, 27]

Several methodological limitations were frequently reported. Many studies used small sample sizes, often fewer than 30 participants, particularly in anesthesia or exploratory settings. Small cohorts limit statistical power and increase the risk of overfitting. The absence of external validation datasets further restricts generalizability across diverse populations and clinical conditions [5, 14, 23]. Improvements in dataset size, population diversity, and multicenter designs are necessary to strengthen reproducibility [2, 9, 11, 15].

Semi-supervised learning strategies were increasingly used to improve generalization by leveraging small labeled datasets with larger pools of unlabeled data. Nonetheless, interpretability remains limited. Although some studies integrated explainability methods, their adoption was relatively low [14, 23]. Transparency in deep learning is essential for clinician trust and safe implementation in clinical workflows.

Future research should focus on building multimodal datasets that integrate cardiovascular, neural, and behavioral signals, developing standardized data collection protocols, and expanding open-access repositories. The integration of explainable artificial intelligence methods is essential for transparency and clinical acceptance [3, 9, 15, 18, 20].

Only a small number of studies assessed the clinical impact of these technologies on patient-centered outcomes such as mortality, length of stay, or quality of life. Ethical and regulatory considerations were also seldom addressed. Huo et al. [5], Kim et al. [17], and Fernández Rojas et al. [22] emphasized the need for regulatory frameworks to ensure the safe and equitable adoption of deep learning–based monitoring systems in clinical environments.

## Discussion

This scoping review synthesized **27 studies** evaluating deep learning models applied to noninvasive physiological signals for the early detection of pain, physiological stress, and hemodynamic deterioration in various clinical environments. The collective findings show rapid growth in this field from 2019 onward, with major contributions from East Asia, Oceania, and North America [1, 4, 9, 14, 18]. The increasing participation of interdisciplinary teams integrating medicine, biomedical engineering, computer science, and physiology has accelerated the development of advanced digital health monitoring tools. These models frequently outperform conventional subjective assessment methods, particularly in high-demand clinical contexts where continuous and objective monitoring is required [3, 5, 11, 15, 20].

A consistent pattern across the included studies is the use of deep learning architectures—such as convolutional neural networks, long short-term memory networks, and transformer-based models—to interpret physiological signals in environments where subjective evaluation is limited or impossible. These models demonstrated the ability to identify subtle physiologic signatures associated with pain, stress, hypotension, respiratory compromise, or impending deterioration. Several studies also reported predictive capabilities minutes or hours in advance of clinical recognition, which has important implications for improving patient outcomes in intensive care, perioperative care, and emergency medicine [4, 5, 13, 19, 23].

Another key trend is the transition from unimodal to multimodal systems. Models that integrate cardiac, neural, behavioral, acoustic, or respiratory information offer more robust performance and reduce susceptibility to noise, artifacts, and confounding factors. Studies combining facial expressions with acoustic or physiological features showed improved diagnostic precision compared with single-signal approaches [5, 12, 22]. Evidence suggests that multimodal integration enhances sensitivity and specificity by providing a richer representation of patient state [20, 21].

Our synthesis aligns with findings reported in the *Journal of Medical Systems*. Burdick et al. [34] demonstrated that multisensory alarms incorporating haptic, auditory, and visual channels outperform traditional alarm modalities. Sangari et al. [35] highlighted the value of spatiotemporal multimodal alarm integration in ICU environments, improving decision-making efficiency. These insights reinforce the potential of multimodal deep learning models for early detection of physiological abnormalities.

Overall, the evidence suggests that deep learning applied to noninvasive monitoring is entering a stage of increasing technical maturity. However, progress is uneven due to persistent challenges related to sample size, clinical validation, generalizability, interpretability, and regulatory frameworks. Addressing these barriers will require coordinated research efforts focused not only on performance metrics but also on clinical safety, transparency, ethical use, and human-centered implementation [6, 7, 13, 24, 28].

## Implications in Clinical Practice of the Use of Physiological Sensors and Deep Learning Models

From a clinical perspective, deep learning models demonstrate potential to anticipate hypotension, respiratory deterioration, or cardiac arrest, sometimes hours before the event becomes clinically apparent [25, 29]. These early warning systems support timely intervention and reduce the risk of adverse outcomes. Additionally, the ability to detect pain or stress in noncommunicative, sedated, or intubated patients represents an important advancement for humane and continuous monitoring [16, 17, 21]. These tools complement clinical judgment and may reduce reliance on subjective assessments.

### Public Health Implications of Artificial intelligence-assisted Physiological Monitoring

Artificial intelligence–assisted physiological monitoring may reduce disparities in access to timely diagnosis and care, particularly in resource-limited regions. Portable or telemetric noninvasive systems could support remote monitoring in rural or home settings [11, 14, 22]. These technologies have the potential to optimize resource allocation, decrease complications related to delayed recognition of deterioration, and improve healthcare system efficiency.

### Barriers and Limitations to Clinical Implementation of Deep Learning Models with Non-Invasive Sensors

Despite promising results, several barriers limit widespread implementation. Many studies rely on small datasets, which restrict model generalizability and increase susceptibility to overfitting [3, 6]. Few studies conducted external or real-time validation, which is necessary to confirm performance across diverse populations and clinical workflows [9, 10]. Additionally, differences in monitoring equipment, preprocessing pipelines, and clinical conditions affect reproducibility.

Model interpretability remains a critical barrier. Only a minority of studies applied explainability methods such as SHAP, attention mechanisms, or feature attribution methods [14, 23]. Limited interpretability reduces clinician trust and raises concerns regarding accountability, particularly for high-stakes predictions related to hemodynamic or respiratory deterioration.

### Ethical, Legal, and Regulatory Considerations in the Clinical Application of Deep Learning in Healthcare

Most studies acknowledged the lack of standardized regulatory frameworks for artificial intelligence in healthcare [7, 13, 28]. This gap generates uncertainty regarding safety requirements, validation standards, and algorithmic auditing. Ethical considerations such as data privacy, algorithmic bias, and equitable access were seldom addressed in detail. Collaborative efforts involving regulatory agencies, health institutions, and multidisciplinary research teams are required to establish clear guidelines for safe and fair integration of AI technologies.

### Recommendations and Future Directions for the Effective Integration of Deep Learning in Clinical Monitoring

Future research should prioritize multicenter studies with diverse populations, since most current evidence derives from single-center or homogenous cohorts [6, 9, 18, 24]. Establishing open-access multimodal datasets and standardized data collection protocols would improve reproducibility and accelerate model development. Prospective and real-time validation in clinical workflows is essential to confirm clinical utility [10, 14, 28].

Explainability must also be strengthened. Although some studies used SHAP or attention-based approaches, their adoption remains limited [25, 26]. Emphasizing XAI methods will be essential to improve transparency, clinician confidence, and clinical acceptance.

Clear regulatory standards are necessary to define minimum requirements for algorithm transparency, external validation, ethical safeguards, and legal accountability [7, 13, 18]. Training programs for clinical personnel should also be implemented to ensure safe and informed use of AI-assisted monitoring systems [8, 12, 25].

Future work should also evaluate real-world outcomes such as reduction of adverse events, improved patient safety, mortality, length of stay, and cost-effectiveness. Following the direction proposed by Burdick et al. [34] and Sangari et al. [35], future research should focus on interpretable multimodal systems capable of integrating spatial, temporal, and behavioral information to enhance early detection of physiological dysregulation.

### Limitations of the Study

This review has several limitations. The search was restricted to three databases and to studies published in English and Spanish, which may have excluded relevant articles published in other languages [11]. In addition, no formal assessment of methodological quality was performed, which limits the ability to determine the strength of the evidence and the level of certainty associated with the findings [4, 7]. Since this is a scoping review, the results should be interpreted as a broad descriptive mapping of the existing evidence rather than definitive conclusions regarding effectiveness or causality [16, 22]. Finally, because artificial intelligence and biomedical signal analysis evolve rapidly, studies published after the final search date may include important findings that were not captured in this review [9, 20].

## Conclusions

This review summarizes the current evidence on noninvasive physiological sensors combined with deep learning algorithms for the early detection of pain, stress, and hemodynamic deterioration in clinical settings. The included studies demonstrate the potential of these technologies to enhance clinical monitoring by improving diagnostic accuracy, anticipating adverse events, and enabling continuous and objective evaluation, particularly in patients who are unable to communicate or in whom subjective assessment is unreliable.

Despite these promising findings, important challenges remain for successful clinical implementation. These include the need for external validation, the incorporation of explainability methods to improve transparency and clinician confidence, and the development of regulatory frameworks to ensure safe and effective integration of artificial intelligence into healthcare. Addressing these challenges will require real-time clinical validation, appropriate training for healthcare personnel, and the development of transparent, explainable, and ethically sound artificial intelligence systems.

## Data Availability

No datasets were generated or analysed during the current study.
